# Effects of High Salinity on Alginate Fouling during Ultrafiltration of High-Salinity Organic Synthetic Wastewater

**DOI:** 10.3390/membranes11080590

**Published:** 2021-07-31

**Authors:** Weiwei Cai, Qiuying Chen, Jingyu Zhang, Yan Li, Wenwen Xie, Jingwei Wang

**Affiliations:** 1 School of Chemistry and Chemical Engineering, Beijing Institute of Technology, Beijing 102488, China; qiuychen@126.com (Q.C.); 13388008290@163.com (J.Z.); bitlybb@163.com (Y.L.); a946682598@163.com (W.X.); 2 School of Environment, Beijing Normal University, Beijing 100875, China; wangjw@bnu.edu.cn

**Keywords:** membrane fouling, high-salinity organic wastewater, alginate, ultrafiltration, multivalent ions

## Abstract

Ultrafiltration is widely employed in treating high-salinity organic wastewater for the purpose of retaining particulates, microbes and macromolecules etc. In general, high-salinity wastewater contains diverse types of saline ions at fairly high concentration, which may significantly change foulant properties and subsequent fouling propensity during ultrafiltration. This study filled a knowledge gap by investigating polysaccharide fouling formation affected by various high saline environments, where 2 mol/L Na^+^ and 0.5–1.0 mol/L Ca^2+^/Al^3+^ were employed and the synergistic influences of Na^+^-Ca^2+^ and Na^+^-Al^3+^ were further unveiled. The results demonstrated that the synergistic influence of Na^+^-Ca^2+^ strikingly enlarged the alginate size due to the bridging effects of Ca^2+^ via binding with carboxyl groups in alginate chains. As compared with pure alginate, the involvement of Na^+^ aggravated alginate fouling formation, while the subsequent addition of Ca^2+^ or Al^3+^ on the basis of Na^+^ mitigated fouling development. The coexistence of Na^+^-Ca^2+^ led to alginate fouling formed mostly in a loose and reversible pattern, accompanied by significant cracks appearing on the cake layer. In contrast, the fouling layer formed by alginate-Na^+^-Al^3+^ seemed to be much denser, leading to severer irreversible fouling formation. Notably, the membrane rejection under various high salinity conditions was seriously weakened. Consequently, the current study offered in-depth insights into the development of polysaccharide-associated fouling during ultrafiltration of high-salinity organic wastewater.

## 1. Introduction

High-salinity organic wastewater, which refers to organic wastewater with salt content higher than 1%, is increasingly produced in recent years, especially from seafood processing, electroplating, petroleum industries, etc. [1,2,3]. The discharge of such high-salinity wastewater may pose seriously negative impacts to aquatic and terrestrial ecosystems [4]. However, the current effective techniques to treat high-salinity organic wastewater are still limited, as the highly saline environment hinders the biodegradation process and causes the invalidation of adsorption due to the presence of numerous ions [5]. Recently, membrane-based technologies have gained broad applications in large-scale water and wastewater treatment processes, attributed to their unique features of superb permeate quality, energy efficiency, small footprint, etc. [6]. Despite not being appropriate for removing ions, ultrafiltration (UF) and microfiltration (MF) have received increasing attention in current years for decontamination of high-salinity organic wastewater for the purposes of removing microbes, particles and various organics [7,8,9]. Membrane fouling is a great concern which hampers the sustainable operation of a membrane system and increases the cost of treatment [10,11,12,13,14], although diverse fouling control methods have been proposed and developed to tackle fouling issues [15,16,17,18,19].

The composition of salts in high-salinity wastewater commonly consists of Na^+^, Ca^2+^, Mg^2+^, Al^3+^, etc., at very high concentrations, of which the main component should be regarded as NaCl. Polysaccharides are considered the typical contaminant present in waste and wastewater. In common aquatic systems such as surface water or general wastewater, many studies have been conducted to explore the influence of ions on organic fouling development, and have found that the presence of ions, especially multivalent ions, strikingly influenced membrane fouling caused by polysaccharides as well as membrane rejection [20,21,22,23]. It was reported that the presence of Ca^2+^ could promote binding between alginate chains, leading to accelerated membrane fouling [23,24].

Unfortunately, most of the existing studies in the literature were conducted under relatively low-saline conditions (e.g., salinity within 1 g/L NaCl), and extremely low multivalent ion concentrations, commonly in the level of mmol/L. There is still a lack of understanding of the effects of high salinity on membrane fouling formation, which is of great significance for ultrafiltration of ionic-based wastewaters such as pharmaceutical wastewater. Our recent work showed that high-concentration Na^+^ significantly increased fouling formed by humic acid, while decreasing fouling by bovine serum albumin [25]. However, the impacts of a high salinity environment including Na^+^, Ca^2+^ and Al^3+^ on polysaccharide fouling were still elusive. It can be expected that such high-concentration ions might exert considerable impacts on the properties and fouling propensities of polysaccharide. In addition, the coexistence of various ions (e.g., Na^+^-Ca^2+^ or Na^+^-Al^3+^) might result in a more complex fouling structure developing. The examination of these hypotheses was the goal of the current work.

Therefore, the current study aims to (i) understand the effects of high salinity on polysaccharide fouling formation, where 2 mol/L Na^+^ was employed to simulate a high saline environment; (ii) reveal the influences of the coexistence of Na^+^-Ca^2+^ or Na^+^-Al^3+^ on polysaccharide fouling, based on the practical composition of high-salinity wastewater; (iii) evaluate the effects of high salinity on membrane retention during ultrafiltration of polysaccharide-associated wastewater.

## 2. Materials and Methods

### 2.1. Experimental Materials

A high-salinity environment was simulated by using NaCl, CaCl_2_ and AlCl_3_, which were all purchased from Macklin Biochemical Company of China. Sodium alginate, which was selected as a surrogate of polysaccharide in wastewater, was purchased from Beijing Solibal Technology Company of China. A raw solution with a concentration of 2500 mg/L was prepared by disintegrating 2.5 g alginate in 1000 mL of deionized water while stirring for 24 h, followed by adjusting the solution pH to 7.0 by using 0.01 mol/L of either NaOH or HCl. The raw alginate solution was stored in a black box at 4 °C and diluted to 50 mg/L with various saline solutions before usage.

### 2.2. Membrane Filtration Experiments

A standard cross-flow membrane filtration setup was used for UF filtration tests in order to evaluate the fouling propensities of alginate under the effects of high salinity conditions. The feed solution was pumped by a digital gear pump (ColeParmer, Vernon Hills, IL, USA) to a commercial crossflow cell (CF042 membrane cell, Sterlitech, Auburn, WA, USA) at a constant flowrate of 0.3 L/min and superficial liquid velocity of 0.1 m/s. The experiments were carried out in a constant pressure mode with the transmembrane pressure remaining at 2 bar. The high-salinity environment was first simulated by Na^+^ alone at a concentration of 2 mol/L (i.e., 11.7% NaCl), after which the effects of coexistence of Na^+^-Ca^2+^ or Na^+^-Al^3+^ were determined by adding 0.5–1.0 mol/L Ca^2+^ or Al^3+^ (i.e., 5.6–11.1% CaCl_2_ or 6.7–13.4% AlCl_3_) to the 2 mol/L Na^+^ solution. A commercial polyethersulfone (PES) UF membrane (Beijing Saipuruite Company, Beijing, China) with a nominal molecular weight cutoff (MWCO) of 50 kDa and effective surface area of 0.0042 m^2^ was used for filtration evaluations. Prior to usage, the PES membrane was immersed in deionized water for around 24 h for wetting purposes and subject to pre-compaction by filtrating deionized water at a pressure of 2 bar before each filtration. The permeate flux was determined according to the rate of change of the permeate mass by an electronic balance (Mettler Toledo, Greifensee, Switzerland) and the data were automatically recorded by LabVIEW software at 5 min intervals. To avoid a change in feed concentration, the permeate was poured back to feed at a fixed interval of 5 min. The filtration experiment finished after operation for 180 min, which allowed all of the flux curves to level off.

### 2.3. Modelling of Membrane Filtration

After each experiment, the total membrane filtration resistance (*R*_t_) was measured according to the following equation:

(1) $$J=\frac{\Delta P}{\;\mu R_{t}}$$

where *J* is the permeate flux in units of m^3^/(m^2^·s); ΔP is the applied transmembrane pressure (TMP) in units of Pa; μ is water viscosity (Pa·s).

The contribution of the fouling resistances, i.e., the intrinsic membrane resistance (*R*_m_), reversible (*R*_r_) and irreversible (*R*_ir_) membrane fouling resistances, was determined according to the resistance-in-series model:

*R*_t_ = *R*_m_ + *R*_r_ + *R*_ir_ (2)

where *R*_m_ was determined by filtering deionized water through the pure membrane. *R*_ir_ and *R*_r_ were evaluated by the following protocol:

(i) The fouled membrane was rinsed using deionized water for around 2 min to remove the reversible fouling layer;
(ii) The value of *R*_m_ + *R*_ir_ was then determined from Equation (1) by filtering deionized water through the rinsed membrane;
(iii) The values of *R*_r_ and *R*_ir_ were then obtained respectively from Equation (2), based on the determined *R*_t_, *R*_m_, and *R*_m_ + *R*_ir_ values.

The achieved flux decline curves were further analyzed using the pore blocking models for crossflow filtration, which were expressed as follows:

Complete blocking model:

(3) $$\frac{dJ}{dt}=-k_{A}\left(J-J^{*}\right)$$

Standard blocking model:

(4) $$\frac{dJ}{dt}=-k_{B}J^{0.5}\left(J-J^{*}\right)$$

Intermediate blocking model:

(5) $$\frac{dJ}{dt}=-k_{C}J\left(J-J^{*}\right)$$

Cake filtration model:

(6) $$\frac{dJ}{dt}=-k_{D}J^{2}\left(J-J^{*}\right)$$

where *k_A_* is the kinetic rate for the complete blocking model in units of min^−1^, *k_B_* (m^−0.5^ min^−0.5^) is the kinetic rate for the standard blocking model, *k_C_* (m^−1^) is the kinetic rate for the intermediate blocking model, and *k_D_* (min m^−2^) is the kinetic rate for the cake filtration model. J* is the flux associated with back-transport due to the liquid shear of the crossflow operation. The best fit model was checked by the relative goodness (*f*) method [26] as shown in Equation (7):

(7) $$f=\frac{{\left(\sigma^{2}\right)}_{min}}{\sigma }$$

where *σ*^2^ is the prediction error square, and (*σ*^2^)*_min_* is the minimum *σ*^2^ value among the four models shown in Equations (3)–(6). The value of *σ*^2^ could be predicted using the following equation:

(8) $$\sigma^{2}=\frac{\sum {\left(Y_{k}-{\hat{Y}}_{k}\right)}^{2}}{n-p}$$

where *Y_k_* and ${\hat{Y}}_{k}\;$represent the experimental and modeled data, respectively. *n*-*p* is the residual degrees of freedom [27].

### 2.4. Other Measurements

In this study, the properties of alginate in terms of zeta potential and particle size were measured using Zetasizer NanoZs (Malvern, Worcestershire, UK). Each measurement was repeated at least six times with the mean value presented as the results. Alginate concentrations in the feed water and permeate were measured with a total organic carbon analyzer (Shimadzu, Japan), and membrane rejection rate was thereby calculated based on the concentration changes between initial feed water and permeate at 180 min. After membrane filtration, the fouled membranes were collected and freeze-dried in a freeze-dryer (Beijing Boyikang Experimental Instrument Company, China) for approximately 12 h. The surface morphology was observed via field emission scanning electron microscopy (FESEM) (Zeiss, Oberkochen, Germany). The functional groups of the fouled membranes were characterized via Fourier transform infrared spectroscopy (FTIR) (Thermo Scientific, Waltham, MA, USA).

## 3. Results

### 3.1. Effects of High Salinity on the Properties of Alginates

**Figure 1:**
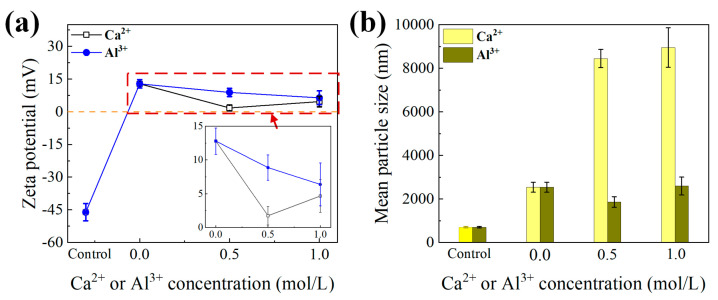
The changes in (**a**) zeta potential and (**b**) particle size of alginates affected by the presence of Na^+^ and different concentrations of Ca^2+^ and Al^3+^. Note: control represents pure alginate solution, whereas 0 mol/L means alginate solution with 2 mol/L Na^+^.

Figure 1 depicts the evolutions of surface zeta potential and particle size of sodium alginate affected by various high-salinity conditions. Compared to previous works [11,22,28], all of the ion additions were investigated at relatively high concentrations (i.e., 2 mol/L Na^+^, 0.5–1.0 mol/L Ca^2+^ and Al^3+^) in order to simulate the real composition of high-salinity wastewater. Obviously, 2 mol/L Na^+^ induced the dramatic increase in zeta potential from −46.13 mV to +12.80 mV, and subsequent addition of Ca^2+^ on the basis of Na^+^ decreased the zeta potential. It is generally acknowledged that the presence of counter ions may increase the zeta potential of organics due to the effects of charge neutralization, and extremely high amounts of ions can exert opposite charges on molecular surfaces [29,30], which explained the sharp rise in zeta potential observed with Na^+^. However, the decrease in positive charges induced by Ca^2+^ probably resulted from the coordination of Ca^2+^ with carboxyl groups in the alginate chains, thus strikingly reducing surface potential to around +2.00 mV. Moreover, it was expected that the changes in surface charge would pronouncedly influence the stability of alginate dispersion, reflected by variations in particle size. Unsurprisingly, Figure 1b displays increased alginate size caused by the presence of Na^+^, probably because of the reduced absolute charge value (Figure 1a) leading to enhanced attraction between alginate molecules. The synergistic influences of Na^+^-Ca^2+^ further enlarged the particle size strikingly, due to the enhanced coordination effects by Ca^2+^ to bridge alginate chains together. Comparatively negligible variations in surface charge and particle size of alginates were found to result from the presence of Na^+^-Al^3+^, which was consistent with a previous study reporting that the binding affinity with alginates was stronger for Ca^2+^ than for Al^3+^ [31].

### 3.2. Effects of High Salinity on Membrane Fouling Propensity

**Figure 2:**
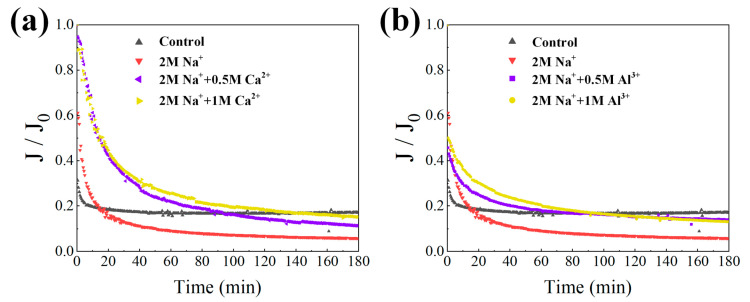
The change in flux profiles against filtration time for alginate solutions affected by the presences of Na^+^, Na^+^-Ca^2+^ (**a**) and Na^+^-Al^3+^ (**b**). Note: control represents pure alginate solution.

The evolution in permeate flux versus time affected by various high-saline conditions is presented in Figure 2. After 180 min filtration, all of the flux profiles nearly reached equilibrium. It was found that the involvement of Na^+^ aggravated alginate fouling formation, witnessed by the lower *J*/*J*_0_ value observed compared with the pure alginate solution. However, the subsequent addition of Ca^2+^ or Al^3+^ mitigated such fouling to some extent. Table 1 displays the membrane fouling resistance values in terms of reversible resistance (*R*_r_), irreversible resistance (*R*_ir_), and intrinsic membrane resistance (*R*_m_). Reversible fouling was derived from the loose attachment of foulants to the membrane surface, which could be removed by physical cleaning methods, while irreversible fouling generally resulted from the formation of a strong matrix of fouling layer or pore narrowing/blocking that could not be removed by physical cleaning [32]. Apparently, the dominant filtration resistance was caused by reversible fouling for all cases studied. Under the condition of simultaneous presence of Na^+^-Ca^2+^, more than 80% of filtration resistance was caused by reversible fouling formation and only 0.18%–0.85% of total resistance was attributed to irreversible fouling. In comparison, the coexistence of Na^+^-Al^3+^ induced more than 20% irreversible fouling formation. This indicated that Ca^2+^ addition led to fouling formation mostly in a loose and reversible pattern, whereas Al^3+^ was beneficial for irreversible fouling development, which inevitably made the membrane difficult to clean. In general, the changes in total resistance were in agreement with the flux profiles in Figure 2, where Na^+^ enhanced membrane fouling formation, while the synergistic effects of Na^+^-Ca^2+^ or Na^+^-Al^3+^ mitigated fouling development.

**Figure 3:**
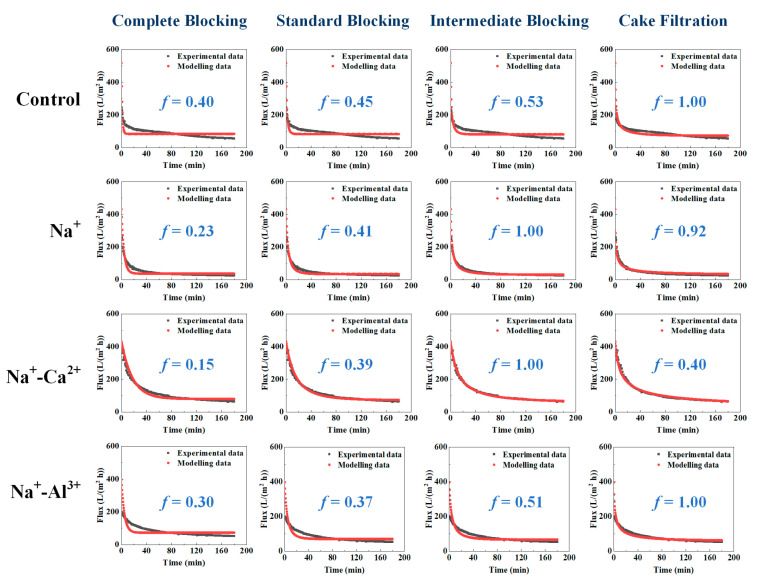
Fitting results of the filtration data with four theoretical models.

To better understand the fouling properties influenced by high salinity, the flux profiles were fitted with the four theoretical fouling models described in Equations (3)–(6). Briefly, the complete blocking model assumed that one foulant clogged and sealed one membrane pore; the standard blocking assumed that the foulant entered into the membrane pores and was adsorbed on the internal walls of membrane pores, causing a decrease in effective pore radius; the cake filtration model assumed that a cake layer was formed on the membrane surface during membrane filtration; the intermediate blocking model assumed the simultaneous occurrence of cake layer formation and complete blocking [33]. Figure 3 exhibits the relative goodness (*f*) results of the modeling, in which the model with *f* value of 1.00 was the best fitting model for the experimental data. Table 2 further depicts the parameters from the best fitting models. The results showed the filtration outcomes of pure alginates and Na^+^-Al^3+^ were best described by the cake filtration model. Despite a relatively higher *k*_D_ value of 5.1 × 10^−6^ min m^−2^ for pure alginates, a larger back-transport flux of 72.93 L/(m^2^ h) was found during filtrating alginates, compared with 66.12 L/(m^2^ h) obtained in the scenario of Na^+^-Al^3+^. Combined with *R*_t_ values in Table 1, it could be speculated that the cake fouling formation was mainly governed by the back-transportation of foulants by liquid shear during crossflow filtration, thus resulting in enhanced fouling when Na^+^ and Al^3+^ simultaneously existed. In contrast, intermediate blocking was found to be the primary fouling mechanism in the presence of Na^+^ alone as well as the coexistence of Na^+^-Ca^2+^, while the fouling formation appeared to be intensified with lower back-transport flux and higher kinetic rate in the presence of Na^+^ alone, which was consistent with the results obtained in Figure 2 and Table 1.

### 3.3. Effects of High Salinity on Membrane Fouling Morphology

**Figure 4:**
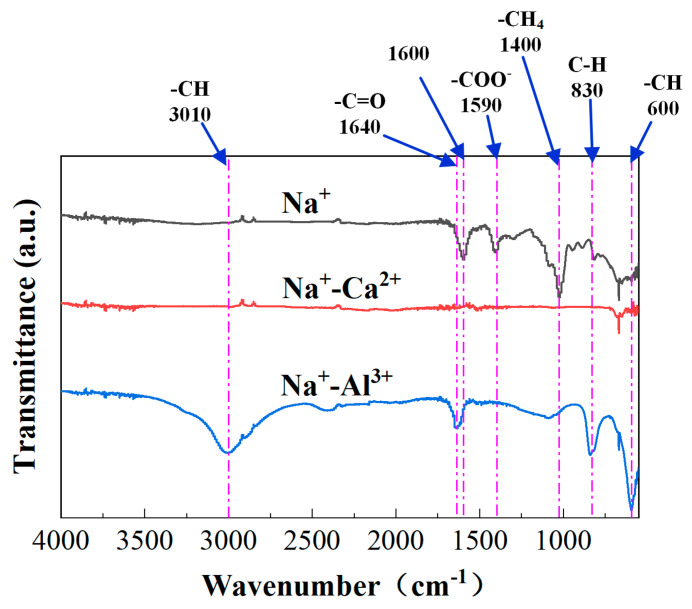
FTIR spectra of the fouled membranes after filtration of alginates under various high-salinity conditions.

**Figure 5:**
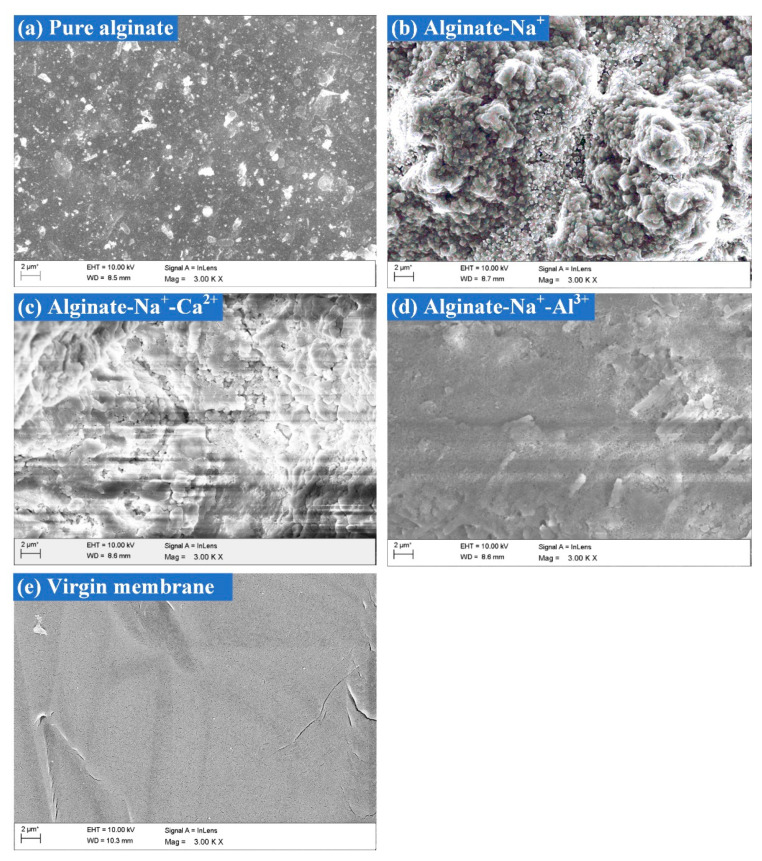
SEM images of fouled membranes after filtration of alginates under various high-salinity conditions. (**a**) Pure alginate; (**b**) Alginate-Na^+^; (**c**) Alginate-Na^+^-Ca^2+^; (**d**) Alginate-Na^+^-Al^3+^; (**e**) Virgin membrane.

After 180 min filtration, the FTIR spectra of the fouled membranes by alginates under various high-salinity environments are depicted in Figure 4. As displayed in Figure 4, the simultaneous presence of Na^+^ and Ca^2+^ showed the smallest FTIR peaks, which indicated that the functional groups were mostly occupied by Ca^2+^. By contrast, further addition of Al^3+^ on the basis of Na^+^ seemed to increase the peak intensities of several functional groups located at the wavenumbers of 3010 cm^−1^, 830 cm^−1^, 600 cm^−1^, etc. As observed in Figure 1b, Ca^2+^ addition induced significant increments in alginate size, probably via bridging effects of Ca^2+^ with carboxyl groups in the alginate chains, and thus negligible FTIR peaks were found in the presence of Ca^2+^. In comparison, the presence of Al^3+^ did not exhibit significant binding with alginates because of the invariable particle size and prominent alginate FTIR characteristic peaks of the fouled membrane upon Al^3+^ addition. However, the increased peak intensities observed implied that the configuration of the alginate cake layer formed might be significantly changed by the involvement of Al^3+^. SEM images of the fouled membranes are displayed in Figure 5. As compared with pure alginates, more serious fouling formation was induced in the presence of Na^+^. When Ca^2+^ was further added, the fouling layer tended to be much smoother with significant defects and cracks appearing on the top, probably explaining the reduced fouling development and resistances shown in Figure 2 and Table 1. In contrast, the fouling layer formed by alginate-Na^+^-Al^3+^ in Figure 5d seemed to be much denser, which might cause the higher degree of irreversible fouling identified under the influence of Al^3+^.

### 3.4. Effects of High Salinity on Membrane Rejection

**Figure 6:**
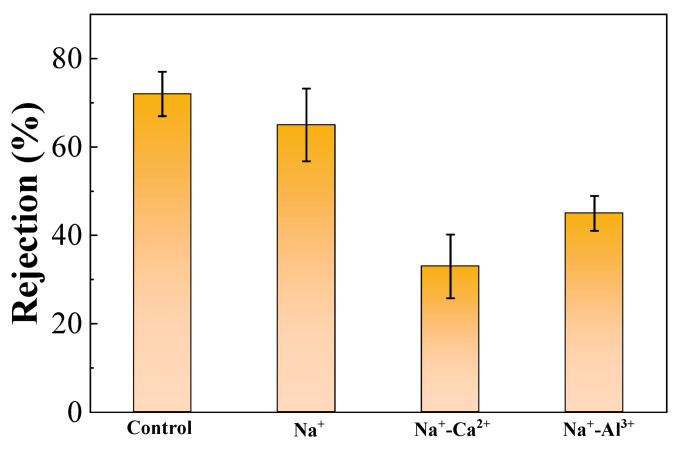
The changes in membrane rejection rates during filtration of alginates affected by various high-salinity environments. Note: control represents pure alginate solution.

Figure 6 exhibits the rejection performance during filtration of alginates affected by various high-salinity conditions. By comparing with initial UF rejection without addition of salts, it was obvious that the membrane rejection rate was strikingly weakened by the high-salinity environments studied. Since our previous study demonstrated that the effects of addition of high-concentration Na^+^ on water flux through a UF membrane could be negligible [25], the principal reason for the reduced membrane rejection may be the changed interactions between foulant–membrane and foulant–foulant instead of salts–membrane. This could be further explained through the fact that the remarkable increase in positive surface charges induced by high salinity (Figure 1a) enhanced the attraction between alginates and the negatively charged UF membrane, thus driving more alginate molecules to pass through the membrane. Notably, a much lower rejection rate of 33% occurred in the presence of Na^+^-Ca^2+^, presumably due to the significant defects and cracks observed in Figure 5c. The higher membrane rejection of 45% found with Na^+^-Al^3+^ might be attributed to the denser cake layer (Figure 5d) and aggravated irreversible fouling formation (Table 1).

## 4. Discussion

The current study systemically investigated the effects of high salinity on fouling behaviors of alginates, during which 2 mol/L Na^+^ as well as 0.5–1.0 mol/L Ca^2+^ or Al^3+^ were employed. It was noteworthy that membrane fouling was enhanced by the presence of Na^+^ compared to pure alginate, probably due to the sharp increase in positive surface charges in the alginate, thus improving the attractions between alginates and the membrane. However, decreased total filtration resistances were observed when Ca^2+^ or Al^3+^ were further added on the basis of Na^+^, and entirely differentiated fouling morphology and foulant properties were found for the cases of Na^+^-Ca^2+^ and Na^+^-Al^3+^. Specifically, Ca^2+^ addition triggered a significant increase in foulant size (Figure 1b) by strongly coordinating with carboxyl groups to bridge alginate chains together, leading to less binding affinity in the formation of the cake layer as well as remarkable defects appearing in the fouling layer (Figure 5c). As was reported in a previous work, alginate can form longer and larger polymeric networks [11]. Larger flocs caught on the membrane surface could generate more porous area than smaller size flocs and decrease the fouling resistance by allowing the water to pass through the floc defects. Thereby, fouling development with the coexistence of Na^+^-Ca^2+^ was mitigated and accompanied by negligible irreversible fouling as compared with Na^+^ alone (Table 1). In contrast, limited binding and ionic bridging effects were found with the coexistence of Na^+^-Al^3+^ (Figure 1b), and increased concentration of organic functional groups was observed (Figure 4), probably due to the changed configuration of the alginate cake layer induced by the involvement of Al^3+^. Eventually, a more compact fouling layer with a higher amount of irreversible fouling was observed with Na^+^-Al^3+^ than the case of Na^+^-Ca^2+^, which presumably led to the higher membrane rejection rate during ultrafiltration of alginates (Figure 6). These results implied that high-saline environments could substantially affect fouling propensities and water treatment effectiveness.

## 5. Conclusions

The current work investigated the impacts of high salinity on membrane fouling by polysaccharides during ultrafiltration of high-salinity organic wastewater. The change in alginate characteristics affected by high-salinity environments, i.e., high-concentration Na^+^ alone, as well as the coexistences of Na^+^-Ca^2+^ and Na^+^-Al^3+^, were examined in depth, with their fouling propensities and retention rates evaluated in a standard crossflow ultrafiltration system. The key findings can be summarized as follows:

(i) Compared with pure alginate, membrane fouling was enhanced by the presence of Na^+^, while decreased fouling development was observed when Ca^2+^ and Al^3+^ were further added on the basis of Na^+^, but entirely differentiated fouling morphology and foulant properties were found with the cases of Na^+^-Ca^2+^ and Na^+^-Al^3+^.
(ii) The membrane rejection rate for organics was generally weakened by high salinity environments; however, the synergistic effects of Na^+^ and Ca^2+^ further worsened permeate quality because of significant defects and cracks appearing on the fouling layer.
(iii) Ca^2+^ enlarged the molecule size of the alginate due to the strong coordination effects of Ca^2+^ with carboxyl groups to bridge alginate chains together, while negligible binding affinity with the alginate was found for Al^3+^ addition.
(iv) Na^+^-Al^3+^ triggered more irreversible fouling formation, probably making the membranes difficult to clean and thus increasing operational cost.
(v) This study gained more insights into the current treatment of high-salinity wastewater using membrane-based techniques.

## Figures and Tables

**Table 1:** Respective resistance of fouled membranes after 180 min filtration. Note: control represents pure alginate solution.

Resistance	Control	Na^+^	Na^+^-Ca^2+^		Na^+^-Al^3+^	
			0.5 M Ca^2+^	1.0 M Ca^2+^	0.5 M Al^3+^	1.0 M Al^3+^
*R*_m_ (×10^12^ m^−1^)	1.61	2.00	1.20	1.71	1.47	1.83
*R*_r_ (×10^12^ m^−1^)	7.58	40.73	9.33	9.53	6.09	9.24
*R*_ir_ (×10^12^ m^−1^)	0.18	2.47	0.09	0.02	2.94	2.86
*R*_t_ (×10^12^ m^−1^)	9.37	45.20	10.62	11.26	10.50	13.90

**Table 2:** Parameters of the best fitting models during membrane filtration of alginate affected by various high-salinity environments.

Salts	Best Fitting Model	Kinetic Rate	Back-Transport Flux (J*, L/(m^2^ h))
Control	Cake filtration	*k*_D_ = 5.1 × 10^−6^ min m^−2^	72.93
Na^+^-Al^3+^	Cake filtration	*k*_D_ = 4.1 × 10^−6^ min m^−2^	66.12
Na^+^	Intermediate blocking	*k*_C_ = 0.0011 m^−1^	30.63
Na^+^-Ca^2+^	Intermediate blocking	*k*_C_ = 0.0002 m^−1^	59.63

## Data Availability

Not applicable.
